# Over Diagnosis or a Hidden Epidemic? The Rising Incidence of Thyroid Cancer in Sri Lanka

**DOI:** 10.1200/GO.22.26000

**Published:** 2022-05-05

**Authors:** Sashiprabha Nawaratne, Janaki Vidanapathirana

**Affiliations:** ^1^ National Cancer Control Programme, Ministry of Health, Colombo, Sri Lanka

## Abstract

Thyroid cancer (TC) is the second commonest cancer among females and the third commonest cancer in both sexes in Sri Lanka. In 2019 the age-standardized rates (ASR) of TC among females and males were greater than the South East Asian estimates and the global estimates of TC for females and males. The aim of the study was to assess the reason for the thyroid cancer burden in Sri Lanka.

**METHODS:**

Secondary data was analyzed from the National Cancer Registry of Sri Lanka. ASR of thyroid cancer was extracted, and age-specific incidence rates were calculated for the respective years. JoinPoint software version 4.9.0.0 was used to analyze trends. Best fit models reported, by Annual Percentage Change and Average Annual Percentage Change (AAPC), 95% CI. A *P* value < .05 were taken as the level of significance.

**RESULTS:**

A total of Total 23,363 TC cases were reported from 2005 to 2019. Among women, ASR has increased threefold from 2005 to 2019 (5.6-17.3) with an AAPC of 7.5% (95% CI, 4.1 to 11.1, *P* < .05) and among males a two-fold rise was seen in ASR (1.7-3.9) with AAPC being 8.0% (95% CI, 5.5 to 10.6, *P* < .05). The highest age-specific incidence rates were seen among women in the reproductive age group (25-34 years). Differentiated thyroid carcinoma comprised 94% (n = 17,861) of all reported TC cases. Papillary TC was the main type of differentiated TC seen in both sexes (n = 14,036).

**CONCLUSION:**

The incidence of TC in Sri Lanka reveals an increasing trend in both sexes. Increase incidence in women in the reproductive age group with a high incidence of papillary TC was highly suggestive of overdiagnosis. However, it cannot be confirmed due to the unavailability of staging and mortality data. Therefore, further studies are recommended to explore the reasons for this observed increase, and the National Cancer Registry should be further strengthened to ensure the completeness of cancer data.

